# Novel Topics in Inflammatory Bowel Disease

**DOI:** 10.1155/2016/8958751

**Published:** 2016-02-23

**Authors:** Atsushi Sakuraba, Alessandro Armuzzi, Kian Keyashian, Makoto Naganuma

**Affiliations:** ^1^Section of Gastroenterology, Hepatology and Nutrition, University of Chicago, 5841 S. Maryland Avenue, MC 4076, Chicago, IL 60637, USA; ^2^IBD Unit, Complesso Integrato Columbus, Gemelli Hospital-Catholic University Foundation, 00168 Rome, Italy; ^3^Division of Gastroenterology, Oregon Health & Science University, Portland, OR 97239, USA; ^4^Department of Gastroenterology and Hepatology, Keio University School of Medicine, Tokyo 160-8582, Japan

We are pleased to announce the publication of the special issue focusing on novel topics in inflammatory bowel disease. It gained the interest of researchers from all over the world and more than 20 articles were submitted for review. Among them, our editorial team consisting of four renowned researchers in this field has selected six articles for publication. This includes basic and clinical research studies as well as translational research focusing on mechanism, epidemiology, treatment outcomes, and genetics. We are confident that this special issue advances the understanding and research of inflammatory bowel disease.

